# IFN-β production induced by PRRSV is affected by GP3 quantity control and CLND4 interaction

**DOI:** 10.1186/s13567-025-01455-6

**Published:** 2025-01-28

**Authors:** Dexin Li, Xinyu Cui, Yingchao Li, Qin Zhang, Hongyan Gao, Youbo Li, Yanmeng Hou, Hongjie Yuan, Yihong Xiao

**Affiliations:** Department of Fundamental Veterinary Medicine, College of Veterinary Medicine, Shandong Agricultural University, 61 Daizong Street, Tai’an, 271018 Shandong China

**Keywords:** CLDN4, GP3, IFN-β, PRRSV, sECL2

## Abstract

Porcine reproductive and respiratory syndrome virus (PRRSV) is one of the most harmful pathogens in the swine industry. Our previous studies demonstrated that the small extracellular domain (ECL2) of CLDN4 effectively blocks PRRSV infection. In this study, we explored the in vivo administration of swine ECL2 (sECL2) and found that it blocked HP-PRRSV infection and alleviated histopathological changes in organs. Notably, sECL2 stimulated cytokine production in the lungs. We observed that CLDN4 upregulated the expression of IFN-β at low doses of GP3. While high doses of GP3 inhibited the activity of the IFN-β promotor, regardless of whether CLDN4 was present. GP3 also downregulated IFN-β by decreasing the phosphorylation of TBK1 and IRF3. These findings highlight functional differences in GP3 under quantity control, which account for the variations in IFN-β induction during the early and late stages of infection. Our results indicate that sECL2 is a promising candidate drug for developing treatments to control PRRS.

## Introduction

Porcine reproductive and respiratory syndrome (PRRS) is one of the most economically significant infectious diseases affecting the global swine industry [1]. The pathogen responsible for PRRS is the PRRS virus (PRRSV), which causes reproductive disorders in pregnant sows and respiratory failure in pigs of all ages [2–4]. The virulence factors of PRRSV isolates vary widely.

Since the first PRRSV (the Lelystad virus) was isolated in 1991, PRRSV has emerged in most countries around the world. In China, after the first case was reported in 1995, a highly pathogenic strain (HP-PRRSV) emerged in 2006, resulting in the deaths of many pigs and causing significant economic losses to the swine industry [5–7]. Since 2017, a NADC30-like strain has gained attention in China [8]. Recombinant viruses originated from the genome of HP-PRRSV and NADC30-like strains are frequently isolated and detected [9]. Unfortunately, there are currently no effective drugs or vaccines to combat PRRS [10], making the development of viable disease control methods a pressing need.

The type I interferon-induced cellular antiviral response serves as the host’s first line of defence against viral infections and is crucial for the innate immune system [11, 12]. PRRSV is known to cause persistent infections and suppress the immune response [2–4]. PRRSV is a single- and positive-stranded RNA virus with a genome of approximately 15.1 kb in length. It belongs to the genus Arteritis within the family *Arteriviridae*. PRRSV contains 10 open reading frames and encodes 14 non-structural and eight structural proteins, which include GP2a, E, GP3, GP4, GP5, ORF5a, M, and N [13–15]. Nsp1, Nsp4, Nsp11, and N antagonize the production of type I interferon, thereby blocking the innate immune response [16–19].

Tight junctions (TJs) serve as the primary barrier between adjacent cell membranes, regulating the flow of ions and solutes while also providing a “fence function” [20]. TJs are composed of membrane proteins such as occludin, claudins (CLDN), and junction adhesion molecules, alongside cytoplasmic proteins such as zonula occludens proteins (ZO-1, ZO-2, and ZO-3) [21, 22].

TJs represent the first line of defence against viral infections, as human and animal viruses employ various strategies to regulate the expression of TJs [23]. Claudins (CLDN) are a family of proteins that play a critical role in the function of TJs, particularly in response to viral infections [24].

Viral infection can either upregulate or downregulate claudin expression, thereby affecting susceptibility to viruses such as human immunodeficiency virus (HIV), hepatitis C virus (HCV), Zika virus (ZIKV), dengue virus (DENV), Japanese encephalitis virus (JEV), severe acute respiratory syndrome coronavirus (SARS-CoV), respiratory syncytial virus (RSV), and others [25–31].

Our previous studies have shown that CLDN4 expression is downregulated in the early stages of PRRSV infection, which enhances viral susceptibility. Our research has revealed that the extracellular domain ECL2 of CLDN4 interacts with the structural protein GP3 of PRRSV, inhibiting viral infection [24].

In this study, the anti-PRRSV role of ECL2 in was evaluated in vivo, focusing particularly on its ability to regulate type I interferon expression.

## Materials and methods

### Cell culture and viruses

African monkey kidney cells (Marc-145) and human embryonic kidney 293 T cells (HEK293T) were cultured in Dulbecco’s modified Eagle medium (DMEM; Gibco, Langley, OK, USA), supplemented with 10% heat-inactivated fetal bovine serum (FBS; Biological Industries, Beit HaEmek, Israel). The cultures were maintained at 37 °C in a humidified incubator with 5% CO_2_.

Porcine pulmonary alveolar macrophages (PAMs) were isolated from the lung lavage of five-week-old piglets free from PRRSV and antibodies against it. These PAMs were maintained in RPMI 1640 medium (Gibco, Langley, OK, USA) supplemented with 15% FBS.

The HP-PRRSV strain TA-12 (GenBank number: HQ416720) was previously isolated and continuously maintained in our lab.

### Plasmids

Total RNA was extracted from Marc-145 cells, and CLDN4 was amplified using reverse transcription–polymerase chain reaction (RT-PCR). The amplified product was then cloned into a p3 × FLAG-myc-CMV^™^-26 vector (Sigma, USA) and confirmed through sequencing and western blot analysis. All constructs were prepared using molecular cloning technology and validated by sequencing. The primer sequences for qPCR are provided in Table 1. The construction of PRRSV (GenBank number: HQ416720) GP2, GP3, GP4, GP5, M, and N gene plasmids with pEGFP-C1 has been described previously [24].

**Table 1:** **Nucleotide sequences used in this study**

Vectors	Gene names	Forward (5′-3′)	Reverse (5′-3′)
p3 × FLAG	Vector	CTTGCGGCCGCGAATTCATCGATAG	CTTGTCATCGTCATCCTTGTAATC
	CLDN4	ACAAGGATGACGATGACAAGATGGCCTCCATGGGGCT	GATGAATTCGCGGCCGCAAGTTACACGTAGTTGCTGGCAGCA
	mIFN-α	GGCTTGACACTCCTGGTACAAATGAG	CAGCACATTGGCAGAGGAAGACAG
	mIFN-β	GGAGATGACGGAGAAGATGC	CCCAGTGCTGGAGAAATTGT
	mIFN-γ	GAGACCATCAAGGAAGACATT	CGACAGTTCAGCCATCAC
	mIL-6	GAAGATTCCAAAGATGTAGCC	GTCAATTCGTTCTGAAGAGG
	mIL-8	CGTACTCCAAACCTTTCCA	CCACTCTCAATCACTCTCAG
	mCLDN4	CTCGTCATCATCAGCATCA	GGCAGAGTAAGGCTTGTC
	mGAPDH	ACCCACTCTTCCACCTTCGACGCT	TGTTGCTGTAGCCAAATTCG
	sTNF-α	GCACTGAGAGCATGATCCGAGAC	CGACCAGGAGGAAGGAGAAGAGG
	sIFN-α	CTGCTGCCTGGAATGAGAGCC	TGACACAGGCTTCCAGGTCCC
	sIFN-β	AGCACTGGCTGGAATGAAAC	TCCAGGATTGTCTCCAGGTC
	sIFN-γ	TGTACCTAATGGTGGACCTC	TCTCTGGCCTTGGAACATAG
	sIL-1β	GCAATAAACAACTTTGGATGGG	GAGTGCTCAAAACGAAGACGA
	sIL-6	GTCGAGGCTGTGCAGATTAGTACC	TTCATCCACTCGTTCTGTGACTGC
	sIL-8	TCCAAACTGGCTGTTGCCTT	ACAGTGGGGTCCACTCTCAA
	sIL-10	ACCTCGACACCGACTACTGCTTC	CCATATAACCTTTGGGTTCGTGGA
	sCLDN4	GTGCTAGGTGTGCTGCTGTC	ATTGTGGGCGGTCCAGG
	PRRSV N	AGATCATCGCCCAACAAAAC	GACACAATTGCCGCTCACTA
	sGAPDH	CCTTCCGTGTCCCTACTGCCAAC	GACGCCTGCTTCACCACCTTCT

		Sense	Anti-sense
siRNA	CLDN4	CGCACAGACAAGCCUUACUTT	AGUAAGGCUUGUCUGUGCGTT
	NC	UUCUCCGAACGUGUCACGUTT	ACGUGACACGUUCGGAGAATT

m: monkey, s: swine

### Transfections

According to the manufacturer’s instructions, Marc-145 cells were seeded in 12-well plates and transfected with the specified plasmids using Lipofectamine 3000 transfection reagent (Invitrogen, USA). The cells were harvested 24 h post-transfection for various analyses.

For the RNA interference experiment, small interfering RNA targeting CLDN4 (siCLDN4) and a scrambled control siRNA (siNC) were synthesized by GenePharma (Shanghai, China) (Table 1). According to the manufacturer’s protocol, Marc-145 cells grown in 12-well plates were transfected with 10 mM siCLDN4 using Lipofectamine RNAiMAX (Invitrogen, USA). At 24 h post-transfection, the cells were collected, and the knockdown efficiency was assessed using qPCR.

### Antibodies

Anti-α glyceraldehyde 3-phosphate dehydrogenase (GAPDH) (AT0002), anti-GFP (AT0028), and anti-FLAG (AT0022) antibodies were obtained from CMCTAG (Dover, DE, USA). Antibodies of anti-TBK1 (1:1000) (3504 s), anti-pTBK1 (5384 s), anti-IRF3 (4302 s), and anti-pIRF3 (4947 s) were obtained from Cell Signaling Technology. The anti-CLDN4 antibody (ab53156) was acquired from Abcam PLC (Cambridge, UK). A monoclonal antibody against PRRSV nucleocapsid (N) protein 6D10 was prepared in our laboratory [32]. Additionally, horseradish peroxidase (HRP)- conjugated anti-mouse and anti-rabbit secondary antibodies were purchased from Jackson Laboratories (West Grove, PA, USA).

### qPCR

Total RNA was extracted using the RNAiso kit (Takara, Dalian, China). cDNA synthesis was performed using the ReverTra Ace qPCR RT kit (Toyobo, Osaka, Japan) following the manufacturer’s instructions. All qRT-PCR assays were conducted using the SYBR green qPCR master mix (Toyobo, Osaka, Japan) in a Roche LightCycler^®^ 96 qPCR System (Roche, Basel, Switzerland). For relative-quantification PCR, data were normalized based on the level of GAPDH mRNA expression in each sample. Primers were designed to target the detection of the PRRSV N gene, and viral mRNA levels were quantified using absolute qPCR. Each reaction was carried out in triplicate, and the results were expressed as the mean ± standard deviation. The primer sequences used for qRT-PCR are listed in Table 1.

### Luciferase reporter assay

HEK293T cells were seeded into 24-well plates at a cell density of 2 × 10^5^ cells/well, and both viral structural proteins and CLDN4 eukaryotic expression plasmids, as well as pGL3-IFN-β-Luc and pRL-TK, were transfected into the cells using PEI 40000 transfection reagent (Yeasen Biotechnology Co., Ltd., Shanghai, China). The PRL-TK plasmid was used to express Renilla luciferase, which served to normalize transfection efficiency. At 24 h post-transfection, the luciferase activities in the cellular extracts were measured using a Dual-Luciferase Reporter Assay Kit (Yeasen Biotechnology Co., Ltd., Shanghai, China), following the manufacturer's instructions.

### Western blot analysis

Equal amounts of cell lysates were boiled with 5 × loading buffer for 10 min. The samples were then separated by SDS–PAGE and transferred to a polyvinylidene fluoride membrane (Millipore, Darmstadt, Germany). After blocking with 5% BSA for 2 h, the primary antibody was incubated for 2 h. The membrane was then incubated with HRP and a conjugated secondary antibody for 1 h. Proteins were visualized using the Clarity^™^ Western ECL substrate (170–5060; Bio-Rad Laboratories) and detected with a western blot fluorescence imager (Vilber Fusion FX7; Vilber Lourmat, Collégien, France). The density of the protein bands was measured using fusion analysis software within the Vilber Fusion FX7 imaging system, with band densities calculated after subtracting the density of the GAPDH bands.

### Animal challenge

Ten healthy five-week-old piglets (negative for both PRRSV antigens and antibodies) were randomly divided into two groups, with five piglets in each group. Group 1 consisted of piglets infected with PRRSV and injected with a dose of 10 mg/kg of the sECL2 polypeptide (Gene Script, Nanjing, China). Group 2 included piglets that were also PRRSV-infected and received an injection of ddH_2_O as a vehicle group.

The piglets were challenged both intranasally (1 mL) and intramuscularly (1 mL) with the HP-PRRSV TA-12 strain (5 × 10^5^ TCID_50_). Polypeptide injections were administered one day before and one day after infection. Following the infection, the piglets were monitored daily for clinical symptoms and rectal temperature. Serum samples were collected on days 1, 3, 5, 7, and 9 days post-infection (dpi).

Four piglets were necropsied at 7 dpi, and another four were necropsied at 14 dpi, with two piglets per group in both instances. The tissues collected were then separated and analyzed for histopathological observations and qPCR.

### Clinical evaluation

Three clinical conditions—behaviour, respiration, and cough—were evaluated using the previously described scoring method [33]. Each condition was assigned a severity rank using numerical indices that ranged from 1 to 4. For deceased animals, the total score was 12, with each category scoring the maximum of 4 (i.e., behaviour = 4, respiration = 4, and cough = 4).

### Histopathological and immunohistochemical (IHC) analysis

Fixed tissues were embedded in paraffin, and 3 μM sections were prepared. The sections were stained with hematoxylin and eosin following standard procedures and then observed under a microscope.

For immunohistochemical (IHC) analysis, the 3 μM sections were placed on lysine-treated glass slides (Solarbio, Beijing, China). Antigen repair was performed using a solution of sodium citrate and citric acid. Endogenous peroxidase activity was blocked with a 3% hydrogen peroxide solution. After blocking with 5% fetal bovine serum for 2 h, the slides were placed with the monoclonal antibody 6D10 at 4 °C overnight and combined with an HRP-conjugated secondary antibody for 1 h.

After washing the slides three times with PBS (140 mM NaCl, 2.7 mM KCL, 10 mM Na_2_HPO_4_, and 1.8 mM KH_2_PO_4_, pH 7.4), immunocomplexes were detected using a DAB solution (Solarbio, Beijing, China). Finally, the sections were counterstained with hematoxylin.

### Statistical analysis

Statistical analysis was conducted using SPSS 23.0 (SPSS Inc., version 23.0; Chicago, IL, USA). All data are expressed as the mean ± standard deviation from at least three biological replicates (*n*) for each condition. Statistical differences between groups were evaluated using Student’s *t*-tests. *P*-values less than 0.05 were considered to indicate statistically significant differences: ^*^P < 0.05, ^**^P < 0.01, ^***^P < 0.001.

## Results

### High CLDN4 expression levels are related to low PRRSV loading

**Figure 1:**
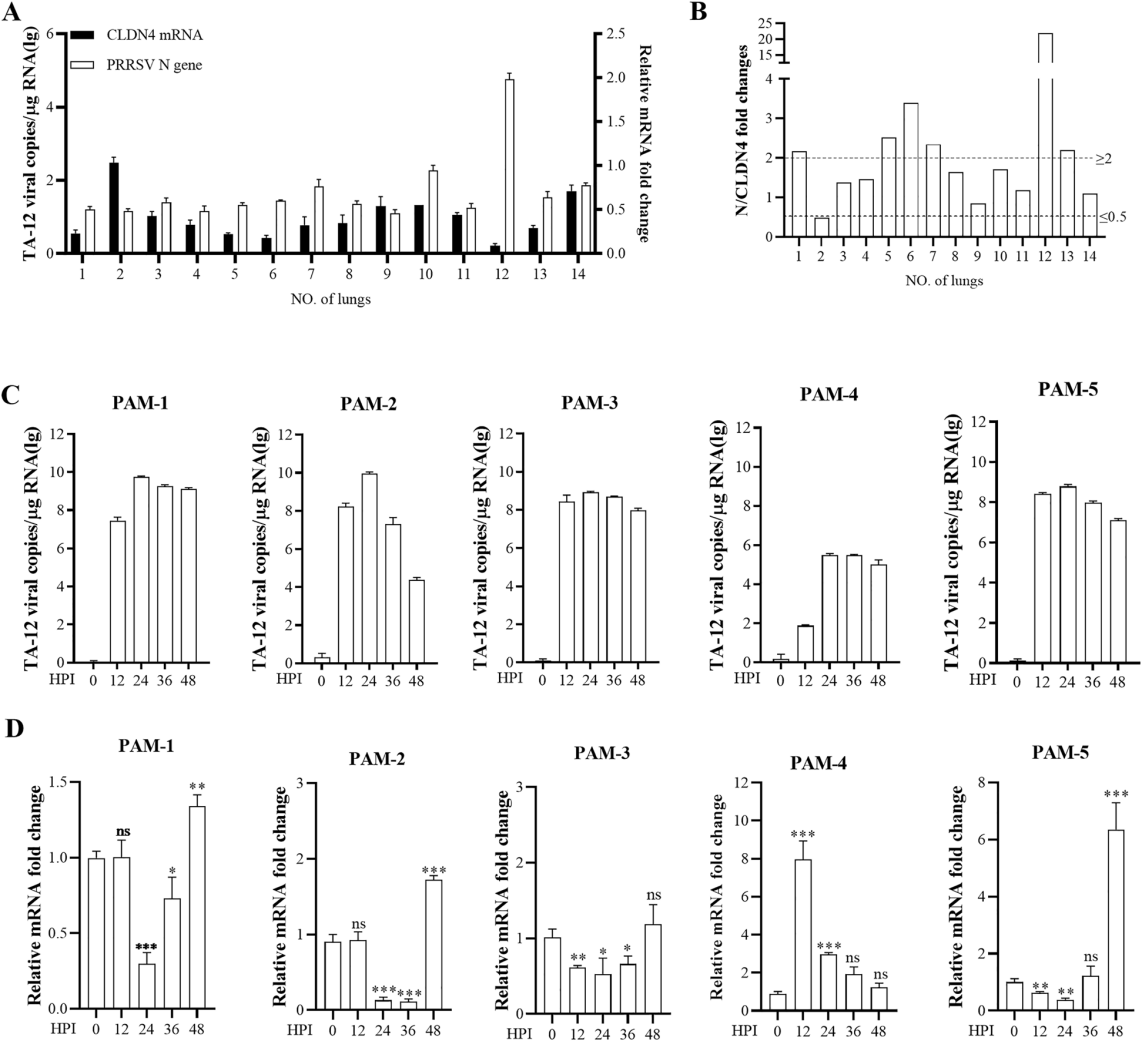
**High CLDN4 expression levels are related to low PRRSV loading.** **A** CLDN4 mRNA levels and PRRSV loading in clinical samples. Total mRNA was prepared from 14 PRRSV-positive lung samples. The viral genome (log copies*g^−1^) and CLDN4 mRNA levels were detected using qPCR. **B** Fold change of N/CLDN4 in (**A**). PAMs were infected with the PRRSV TA-12 strain. Cells were collected at 0, 12, 24, 36, and 48 h post-infection, and total RNA was extracted. The mRNA levels of the PRRSV N gene (**C**) and CLDN4 mRNA (**D**) were measured by qPCR. GAPDH mRNA was used as an internal control. Error bars indicate the SDs of three experimental replicates. Student’s *t*-tests evaluated differences: ***P < 0.001, **P < 0.01, *P < 0.05.

To verify the expression level of CLDN4 during PRRSV infection, we examined PRRSV-positive lung samples from 14 clinical cases (Figure 1A). The results indicated that the viral copy number was negatively correlated with the mRNA level of CLDN4 in more than half of the samples. We also calculated the fold changes of N/CLDN4. The findings showed that the fold changes for samples Nos. 1, 5, 6, 7, 12, and 13 were greater than 2.0 (Figure 1B), while sample No. 2 was below 0.5, indicating significant changes. These results suggest that higher levels of CLDN4 are associated with lower PRRSV infection rates.

To further confirm this relationship, we measured the viral copy number and the mRNA level of CLDN4 in PAM derived from five pigs. The data revealed that in PAM cells (excluding PAM-4), the viral copy number of PRRSV peaked at 24 h post-infection (hpi), coinciding with the lowest expression levels of CLDN4 (Figure 1C). For PAM-4, the lowest viral copies (lg N gene copy number = 1.9, 5.5, 5.5) were observed, along with the highest relative expression level of CLDN4 (relative mRNA fold change = 8.0, 1.9, 1.2) at 12, 24, and 36 hpi. Conversely, PAM-2 exhibited the highest viral copies (lg N gene copy number = 8.2, 10, 7.3) but had relatively low CLDN4 expression levels (relative mRNA fold change = 0.9, 0.1, 0.1) at 12, 24, and 36 hpi.

Overall, these results indicate that CLDN4 expression is negatively correlated with PRRSV infection and may be useful for screening pigs that are resistant to PRRSV.

### sECL2 relieves the symptoms and organ damage caused by PRRSV infection

**Figure 2:**
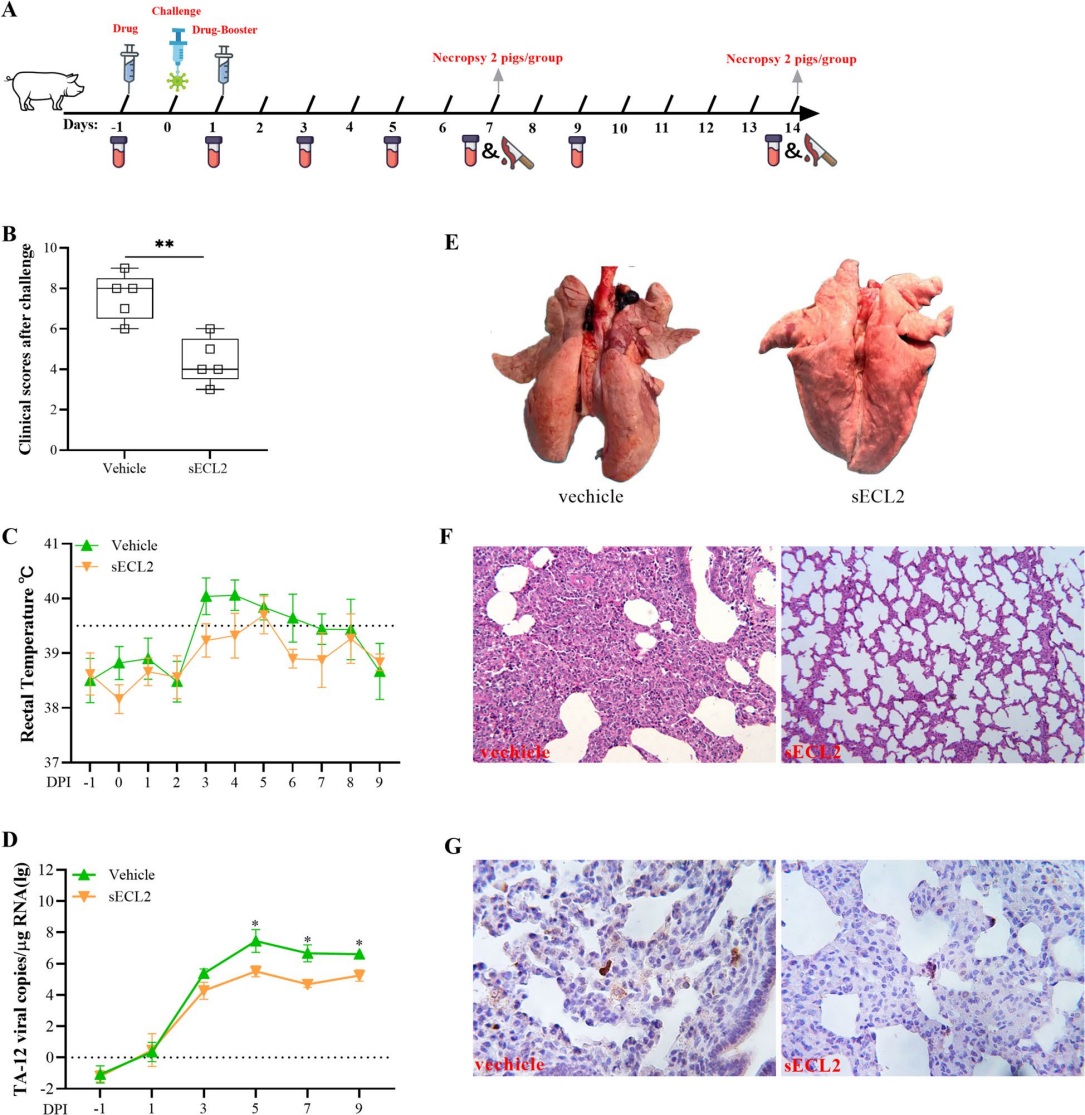
**sECL2 alleviates the pathogenicity caused by PRRSV infection.** **A** Pattern diagram of animal experiments. **B** Records of clinical manifestations and average clinical score for each group after the HP-PRRSV challenge. **C** Changes in daily rectal temperature of piglets infected with HP-PRRSV. A clinical fever was defined as rectal temperature ≥ 39.5 °C. **D** The level of PRRSV mRNA in sera of piglets (log copies*ml^−1^) was detected by qPCR. **E**–**G** Lung gross lesions and histopathological and immunohistochemical examinations (200 ×). Lungs from an HP-PRRSV-infected and vehicle (injected ddH_2_O)-treated control piglets. Lungs from piglets were infected with HP-PRRSV and treated with sECL2. Error bars indicate the SDs of three experimental replicates. Student’s *t*-tests evaluated differences: *P < 0.05.

As previously demonstrated, swine ECL2 (sECL2) can block and neutralize PRRSV infection [24]. To investigate its role in vivo, sECL2 was administrated to PRRSV-infected pigs (Figure 2A). The pigs treated with sECL2 exhibited milder clinical symptoms compared to those in the vehicle group (Figure 2B). The rectal temperature in the sECL2 group increased only at 5 dpi (Figure 2C). More copies of the viral genome were detected in the vehicle group at 3-, 7-, and 9-dpi. (Figure 2D). Histopathological observations revealed a greater number of infiltrated macrophages in the alveolar wall of the vehicle group (Figures 2E and F). Furthermore, higher amount of PRRSV protein was observed in the vehicle group compared to the polypeptide group (Figure 2G). Overall, these results indicate that sECL2 can effectively block PRRSV infection and reduce the damage caused by the virus in vivo.

### CLDN4 is involved in cytokine expression

**Figure 3:**
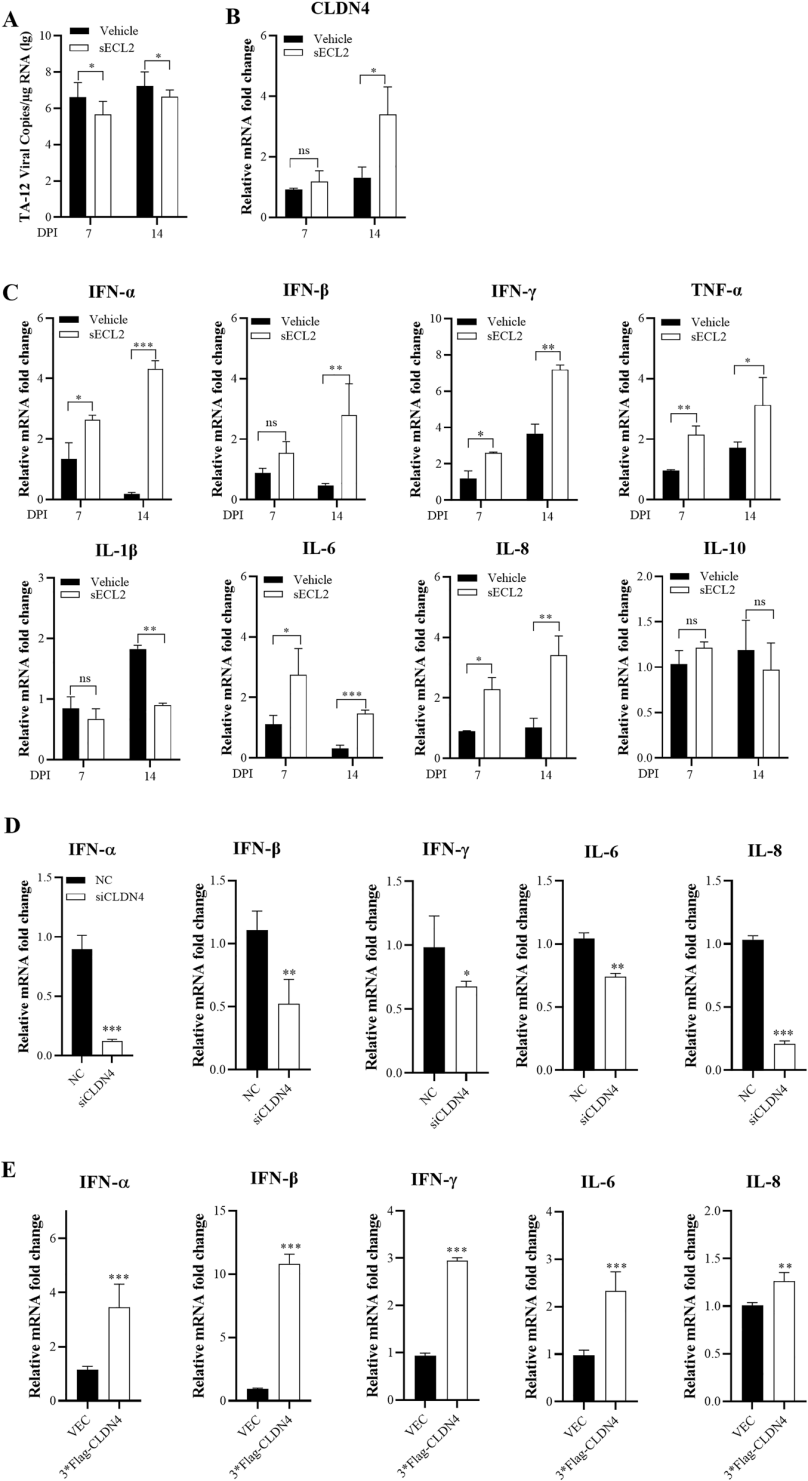
**CLDN4 upregulates cytokine expression.** **A**–**C** The viral genome, CLDN4, and cytokine expression changed after administrating sECL2. Total mRNA of lungs from sECL2 and the control group at 7 and 14 dpi was prepared. q-PCR was used to detect the expression of mRNA of viral load (log copies*g^−1^) (**A**), CLDN4 (**B**), and cytokines including IFN-α, IFN-β, IFN-γ, TNF-α, IL-1β, IL-6, IL-8, and IL-10 (**C**). **D**, **E** CLDN4 upregulates cytokine expression after PRRSV infection. CLDN4 was knocked down with specific siRNA (**D**) or overexpressed by transfection 3*Flag-CLND4 (**E**) in Marc-145 cells. Total cellular RNA was extracted by inoculation with HP-PRRSV (0.1 MOI) 24 h post-infection. q-PCR was performed to verify the IFN-α, IFN-β, IFN-γ, IL-6, and IL-8 mRNA levels. GAPDH mRNA was used as an internal control. Error bars indicate the SDs of three experimental replicates. Student’s *t*-tests evaluated differences: ***P < 0.001, **P < 0.01, *P < 0.05.

It has been reported that PRRSV antagonizes the innate immune system [16–19]. The above results showed that sECL2 can decrease PRRSV infection. We confirmed that the viral load in the lungs of a TA-12-infected pig treated with sECL2 was significantly lower than that in the vehicle group (Figure 3A). Moreover, the mRNA levels of CLDN4 at 14 days post-infection were substantially higher in the sECL2 group compared to the vehicle group (Figure 3B).

Next, we investigated the role of sECL2 in innate immune responses by first studying cytokine expression in the lungs of both the sECL2 and control groups. The results showed that sECL2 upregulated the expression of IFN-α, IFN-β, IFN-γ, TNF-α, IL-1β, IL-6, and IL-8, but not IL-10 (Figure 3C). These findings suggest that sECL2 may be involved in cytokine expression.

We further examined the effect of CLDN4 on PRRSV-induced cytokine changes through in vitro validation. Marc-145 cells were transfected with CLDN4-specific small interfering RNA (siRNA) and control NC siRNA (Figure 3D). The qPCR results showed that the knockdown of CLDN4 significantly downregulated the mRNA levels of IFN-α, IFN-β, IFN-γ, IL-6, and IL-8 compared to the NC. Conversely, the overexpression of CLDN4 led to a significant increase in the mRNA levels of IFN-α, IFN-β, IFN-γ, IL-6, and IL-8. Together, these results indicate that CLDN4 plays a role in regulating cytokine expression.

### GP3 antagonizes the function of CLDN4 in upregulating IFN-β promoter activity

**Figure 4:**
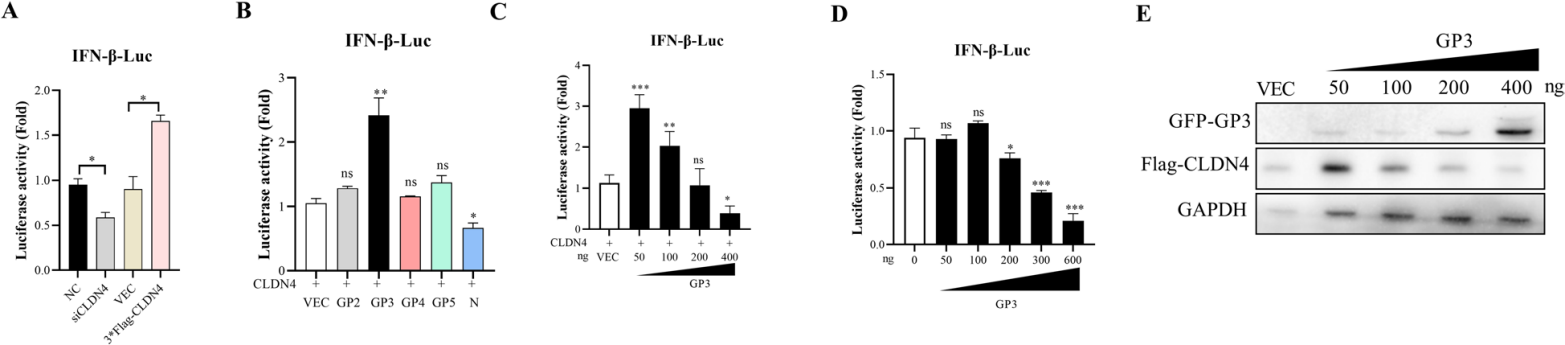
**GP3 is involved in CLDN4-dependent IFN-β promoter activity.** **A** CLDN4 upregulated IFN-β promoter activity. HEK293T cells were co-transfected with siCLND4 or 3*Flag-CLND4, and pGL3-IFN-β-Luc and pRL-TK plasmids. Cells were collected for luciferase analysis after adding SeV for 8 h. **B** GP3 was involved in CLDN4-dependent IFN-β promoter activity. Constructs containing GP2, GP3, GP4, GP5, and N genes were co-transfected with 3*Flag-CLND4, pGL3-IFN-β-Luc, and pRL-TK plasmids into HEK293T cells. Cellular samples were collected after adding SeV for 8 h. Luciferase activity was analyzed for firefly and Renilla luciferase activity using a dual-luciferase reporter assay kit. **C** GP3 regulated the IFN-β promoter activity in a dose-dependent manner in the presence of CLDN4. Recombinant plasmids of GFP-GP3 with 50, 100, 200, or 400 ng and 3*Flag-CLND4 were co-transfected with pGL3-IFN-β-Luc and pRL-TK into HEK-293 T cells for 24 h. After adding SeV for 8 h, cellular samples were collected, and luciferase activity was analyzed for firefly and Renilla luciferase activity using a dual-luciferase reporter assay kit. **D** GP3 downregulated IFN-β promoter activity at high doses. Recombinant plasmids of GFP-GP3 with 50, 100, 200, or 400 ng were co-transfected with pGL3-IFN-β-Luc and pRL-TK into HEK293T cells for 24 h. After adding SeV for 8 h, cellular samples were collected, and luciferase activity was analyzed using a dual-luciferase reporter assay kit for firefly and Renilla luciferase activity. **E** GP3 degraded CLDN4 in a dose-dependent manner. HEK293T cells were co-transfected with 50, 100, 200, or 400 ng of GP3 at different doses with CLDN4 for 24 h. Cellular proteins were prepared and analyzed using a western blot. GAPDH was used as an inner control. Error bars indicate the SDs of three experimental replicates. Student’s *t*-tests evaluated differences: ***P < 0.001, **P < 0.01, *P < 0.05.

The results described in the last section indicate that CLDN4 plays a role in cytokine production and is essential in PRRSV infection. The significant changes observed in the mRNA levels of IFN-α, IFN-β, and IFN-γ mRNA suggest that CLDN4 may be involved in innate immunity. To further investigate these findings, we examined the effect of CLDN4 on the promoter activity of IFN-β. The results demonstrated that CLDN4 knockdown significantly inhibited IFN-β promoter activity compared to the NC, while overexpression of the CLDN4 plasmid significantly enhanced IFN-β promoter activity (Figure 4A). These findings imply that CLDN4 upregulates the promotor activity of IFN-β, thereby increasing IFN-β expression.

To explore the impact of viral proteins on CLDN4 function, we co-transfected viral structural proteins with a construct containing the IFN-β promoter. The results demonstrated that GP3 significantly promoted IFN-β promotor activity when co-transfected with CLDN4 (Figure 4B). We used the nucleocapsid protein N as a control due to its ability to antagonize IFN-β production [34].

To further confirm the role of GP3, we applied gradient doses of GP3. Our findings revealed that a lower dose of GP3 significantly upregulated IFN-β promoter activity, while a higher dose led to the downregulation of IFN-β promoter activity (Figure 4C). Interestingly, the presence of GP3 alone resulted in a dose-dependent downregulation of IFN-β promoter activity (Figure 4D). Western blot results indicated that GP3 reduced the level of CLDN4 in a dose-dependent manner (Figure 4E).

It is known that GP3 downregulates CLDN4 by promoting the ubiquitination of the transcription factor SP1 during the early stages of PRRSV infection [24]. Our study suggests that in the later stages of infection, a higher amount of GP3 downregulates CLDN4, subsequently inhibiting IFN-β promoter activity to facilitate PRRSV infection. These findings indicate that GP3 antagonizes the function of CLDN4 in enhancing IFN-β promoter activity.

### GP3 downregulates IFN-β expression by decreasing the phosphorylation of IRF3 and TBK1

**Figure 5:**
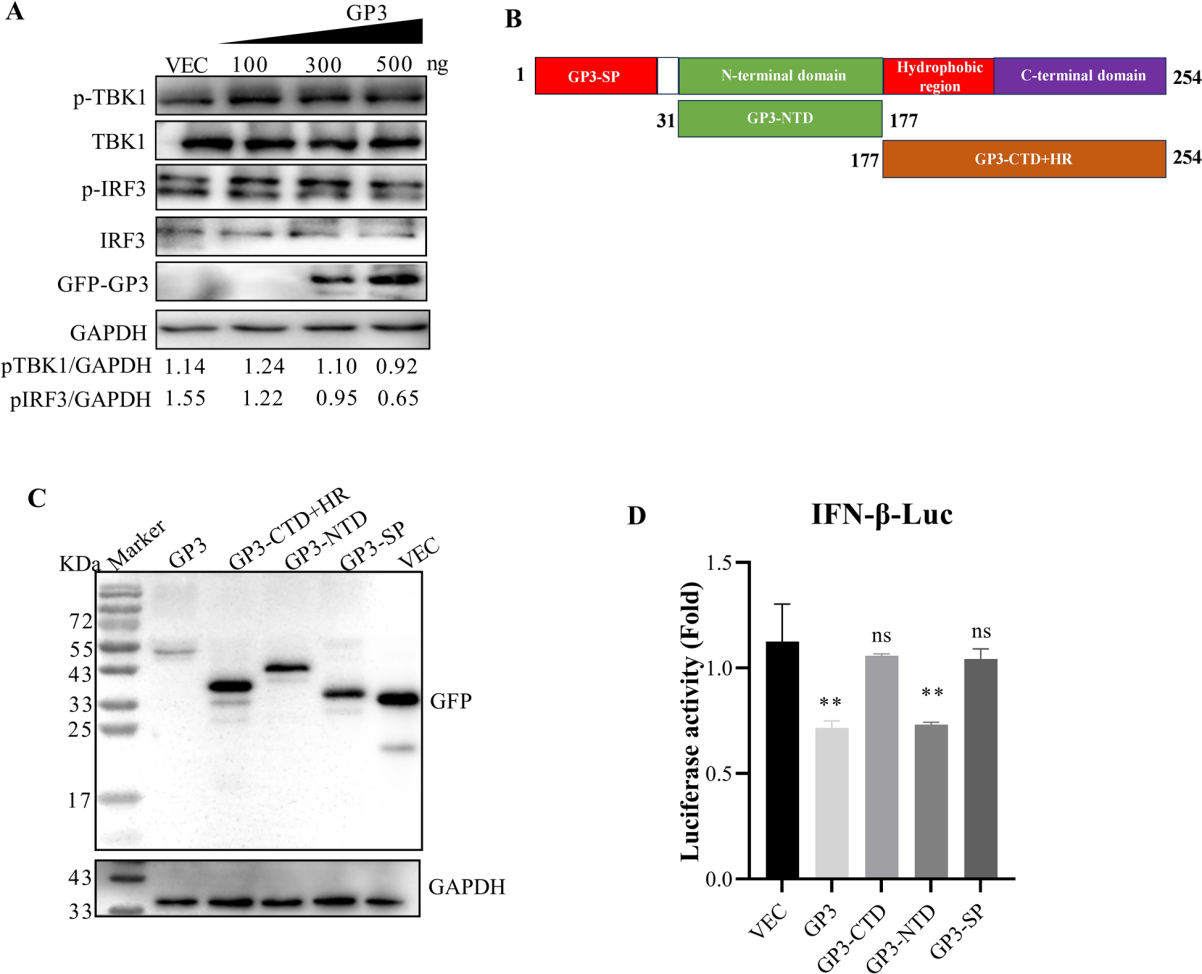
**GP3 inhibits type I interferon production by targeting TBK1 and IRF3.** **A** GP3 decreased the phosphorylation of TBK1 and IRF3. HEK293T cells were transfected with GFP-GP3 of 0, 100, 300, or 500 ng for 24 h. Eight hours before collecting cells, SeV was added. Protein samples were subjected to analysis by western blot. **B** Schematic diagram representing the HP-PRRSV GP3 protein deletion mutant constructs. **C** Identification of truncated GP3 by western blot. HEK293T cells were transfected with plasmids for 24 h and analyzed using a western blot. **D** GP3-NTD was the functional domain for downregulating the promoter activity of IFN-β. The HEK293T cells were co-transfected with truncated GP3 plasmids, pGL3-IFN-β-Luc, and pRL-TK. After adding SeV for 8 h, cellular samples were collected, and luciferase activity was analyzed for firefly and Renilla luciferase activity using a dual-luciferase reporter assay kit. Error bars indicate the SDs of three experimental replicates. GAPDH was used as a loading control. Statistical analysis was performed using Student’s *t*-tests: **P < 0.01.

To investigate the role of GP3 in the expression of IFN-β, we examined the typical TBK1-IRF3 signaling pathway. Our results indicated that GP3 can inhibit the phosphorylation of TBK1 and IRF3 in a dose-dependent manner (Figure 5A). GP3 consists of N- and C-terminal structural domains connected by a short hydrophobic region [35] (Figure 5B). We generated truncated plasmids for further analysis (Figure 5C). HEK293 cells were co-transfected with the GP3-truncated plasmids alongside the pGL3-IFN-β-Luc and pRL-TK plasmids. The findings revealed that GP3-NTD significantly reduced the promoter activity of IFN-β in comparison to cells transfected with the empty vector (Figure 5D). Overall, these results demonstrate that the NTD of the structural protein GP3 significantly reduces IFN-β promoter activity.

## Discussion

Since the emergence of PRRS, several vaccines have been developed to prevent and control it. However, the pathogenesis of PRRSV infection is not yet fully understood, particularly with the advent of mutant strains [36]. It is crucial to investigate the pathogenesis of PRRS further and develop effective vaccines and antiviral medications.

As a crucial component of TJs, CLDN is often hijacked by viruses, either by acting as cellular receptors or prompting the degradation of TJs to facilitate viral entry and transmission. Several drugs targeting TJs have been reported [37–45].

Our previous study found that CLDN4 plays a crucial role in the process of PRRSV infection and that ECL2 is a promising candidate as an anti-PRRSV drug [24]. In this study, we observed that the mRNA level of CLDN4 in the lung tissues of PRRSV-positive pigs is negatively correlated with the viral copy number. Although in individual pigs, the expression of CLDN4 did not correlate with the viral copy number. It was confirmed that PRRSV infection was limited in pigs with high expression levels of CLDN4. Therefore, from this perspective, CLDN4 could be useful for screening PRRSV-resistant pigs.

Interestingly, our findings indicate that CLDN4 is involved at the mRNA level of cytokines that depend on GP3. The co-presence of GP3 and CLDN4 can activate IFN-β promoter activity. However, high doses of GP3 can inhibit the IFN-β promoter’s activity. Low doses of GP3 during the entry stage of PRRSV increase the expression of IFN and prevent virus infection. In contrast, high doses of GP3 reduce IFN expression and facilitate viral infection. These results offer a new explanation for the immunosuppression caused by GP3.

It is reported that TNF-α inhibits the replication of PRRSV [46]. Additionally, HP-PRRSV affects the release of TNF-α stimulated by TLR4 and TLR3 by altering the regulation of ERK. In comparison to less pathogenic strains of PRRSV, HP-PRRSV infection leads to reduced production of TNF-α by PAMs. This reduction may partially explain the pathogenesis of HP-PRRSV [47].

The cellular antiviral response induced by type I interferon-induced serves as the host's first line of defense against viral infections and is essential for the innate immune system [11]. Our research indicates that CLDN4 can activate type I interferon signalling, increasing IFN-β promoter activity (Figure 4).

We demonstrated the critical role of CLDN4 in HP-PRRSV infection in vivo and found that CLDN4 regulates virus-induced innate immunity. However, further investigation is needed to understand how GP3 affects the promoter activity of IFN-β. There are two main considerations here. First, GP3 degrades CLDN4, reducing CLDN4 expression (Figure 5), thereby impacting IFN-β promoter activity. Second, GP3 directly influences the innate immune signaling pathway, which also affects IFN-β promoter activity. Previous studies have not shown that GP3 impacts IFN-β promoter activity to regulate type I interferon production [16, 18]. This finding may be related to the virulence of the different strain, as the GP3 sequence shares only about 55% homology between type I and type II [48]. Alternatively, it could be related to the amount of GP3 present which caused by the infection stages of PRRSV.

Our findings suggest that CLDN4 plays a significant role in the innate immune response against HP-PRRSV infection. Our study offers a theoretical foundation for further understanding the pathogenesis of HP-PRRSV and for the development of antiviral drugs.

## Data Availability

All data generated or analyzed during this study are included in this published article and its supplementary information files.
